# Serum Uric Acid Levels Are Related to Diabetic Peripheral Neuropathy, Especially for Motor Conduction Velocity of Tibial Nerve in Type 2 Diabetes Mellitus Patients

**DOI:** 10.1155/2023/3060013

**Published:** 2023-05-18

**Authors:** Hui Zhang, Carvalho Vladmir, Zhen Zhang, Wan Zhou, Jiang Xu, Wanwan Zhao, Yang Chen, Mengting He, Ya Zhang, Wei Wang, Haoqiang Zhang

**Affiliations:** ^1^Henan Key Laboratory of Rare Diseases, Endocrinology and Metabolism Center, The First Affiliated Hospital and College of Clinical Medicine of Henan University of Science and Technology, Luoyang, China; ^2^Department of Endocrinology, Affiliated Zhongda Hospital of Southeast University, Nanjing, China; ^3^Department of Endocrinology, Division of Life Sciences and Medicine, The First Affiliated Hospital of USTC, University of Science and Technology of China, Hefei, China; ^4^Department of Nephrology, Division of Life Sciences and Medicine, The First Affiliated Hospital of USTC, University of Science and Technology of China, Hefei, China; ^5^Graduate School, Bengbu Medical University, Bengbu, China; ^6^Graduate School, Anhui Medical University, Hefei, China

## Abstract

**Background:**

Oxidative stress is one of the most critical factors that contribute to the pathogenesis of neuronal damage, including diabetic peripheral neuropathy (DPN). Uric acid is a kind of natural antioxidant that plays a major role in the antioxidant capacity against oxidative stress. Here, we aim to determine the role of serum uric acid (SUA) in the DPN of patients with type 2 diabetes mellitus (T2DM). *Patients and Methods*. 106 patients with T2DM were recruited and divided into the DPN group and the control group. Clinical parameters, especially for motor nerve fiber conduction velocity and sensory nerve fiber conduction velocity, were collected. Differences between T2DM patients with and without DPN were compared. Correlation and regression analyses were performed to explore the association between SUA and DPN.

**Results:**

Compare with 57 patients with DPN, 49 patients without DPN showed lower HbA1c and elevated SUA levels. Additionally, SUA levels are negatively associated with the motor conduction velocity of tibial nerve with or without adjusting for HbA1c. Besides, it is suggested that decreased SUA levels may influence the motor conduction speed of the tibial nerve by multiple linear regression analysis. Moreover, we demonstrated that decreased SUA level is a risk factor for DPN in patients with T2DM by binary logistic regression analysis.

**Conclusion:**

Lower SUA is a risk factor for DPN in patients with T2DM. Additionally, decreased SUA may influence the damage of peripheral neuropathy, especially for motor conduction velocity of the tibial nerve.

## 1. Introduction

According to estimates, there are 451 million adults worldwide who have been diagnosed with diabetes. It is projected that by 2045, this number will increase to 693 million [1]. There were approximately 1.09 billion adults in mainland China in 2013. Additionally, the overall prevalence of diabetes of adults was 10.9% [2]. Type 2 diabetes mellitus (T2DM) is characterized by chronic hyperglycemia, insulin resistance and usually along with lipid disorder [3], and hyperuricemia [4]. Increased serum uric acid (SUA) is associated with diabetes [5] and several diabetic complications [6–12] including diabetic peripheral neuropathy (DPN). Indeed, hyperuricemia is common in patients with T2DM and associated with the C-peptide incremental effect of islet beta cell function in T2DM, especially in female patients [13]. Except for diabetes itself, SUA levels are higher in T2DM patients with DPN than those without DPN [14, 15]. It is also suggested that elevated SUA levels increased the chance of developing peripheral polyneuropathy in patients with T2DM [16]. Another Chinese clinical study indicated that there is a significant association between elevated SUA levels and DPN. Additionally, SUA may be a valuable indicator to predict the occurrence of DPN in T2DM patients [17]. However, a large sample study with 2809 individuals found that SUA is not an independent risk factor of DPN [18]. However, Zhuang et al. demonstrated that the low SUA level is closely associated with DPN [19].

DPN is a major risk factor for diabetes-related lower-extremity complications and presents clinically as distal symmetrical sensorimotor polyneuropathy [20]. It affects about 50% of T2DM patients with a diabetic duration of more than 10 years [21]. Additionally, it also appears in newly diagnosed patients with T2DM [22]. As the mechanism is not clear, there is no effective therapy for patients with DPN. Oxidative stress is one of the critical factors that contribute to the pathogenesis of neuronal damage, including DPN [23–25]. Further laboratory experiments demonstrated that oxidative stress is one of the potential mechanisms of DPN [26]. A recent study indicated that oxidative stress is involved in the apoptosis of Schwann cells and takes part in DPN [27]. Thus, it is suggested that reducing of oxidative stress may improve DPN. Interestingly, uric acid is a kind of natural antioxidant and plays a major role in the antioxidant capacity against oxidative stress [28].

In general, SUA may be a double-edged sword in different studies. Although a great number of researches instant that elevated SUA is a risk factor for DPN, low SUA levels may also contribute to DPN in patients with T2DM. These conflicting results have indicated that the relationship between SUA and DPN in patients with T2DM is still unclear and needs further exploration. Here, we performed this study to investigate the relationship between SUA and DPN, in T2DM patients without uric acid treatments.

## 2. Material and Methods

### 2.1. Experiment Design and Ethics

This cross-section study was conducted in The First Affiliated Hospital of University of Science and Technology of China (USTC). 106 patients certificated the standard of T2DM were recruited. In these individuals, 57 patients with T2DM were diagnosed as DPN and 49 T2DM patients without DPN. All participants were informed about the process of this experiment and given a handwritten signature on the informed consent before the experiment. The study was approved by the Ethics Committee of The First Affiliated Hospital of USTC and complied with the Declaration of Helsinki. This study was registered on the Chinese Clinical Trial Registry (ChiCTR2100046905).

### 2.2. Inclusion and Exclusion Criteria

The World Health Organization 1999 [29] criteria for T2DM diagnosis were used for patient recruitment in this present work. Exclusion criteria were the following: any other clinically evident causes of neuropathy apart from diabetes and taking drugs affecting the serum level of SUA such as diuretics, cyclosporine, allopurinol, estrogen, and cytotoxic drugs. DPN patients were diagnosed based on the Toronto consensus of diabetic neuropathy [30]. Patients without DPN were defined as control.

### 2.3. Clinical Data Collection

Age, gender, and education as well as the duration of diabetes mellitus (DM) and the duration of high blood pressure (HBP) were collected. Triglyceride (TG) (Roche Group, Basel, Switzerland; 0.1-10.0 mmol/L), total cholesterol (TC) (Roche Group, Basel, Switzerland; 0.1-20.7 mmol/L), low-density lipoprotein cholesterol (LDL-C) (Ningbo Ruiyuan Biotechnology Co., Ltd., Ningbo, China; 0.2-11.6 mmol/L), high-density lipoprotein cholesterol (HDL-C) (Roche Group, Basel, Switzerland, 0.08-3.88 mmol/L), and SUA (Beckman Coulter, Brea, USA; 89-1785 umol/L) were determined by their kits described above. Additionally, microcolumn ion-exchange chromatography was employed to detect glycosylated hemoglobin (HbA1c) levels. These measurements were conducted in The First Affiliated Hospital of USTC, Center Laboratory for medical usage. These data were collected for further analysis. Body mass index (BMI) was calculated as weight (kg)/height (m)^2^.

### 2.4. Neurophysiological Examinations

All patients underwent neurophysiological examinations by electromyographic evoked potential meter according to the protocol of the manufacturer (Natus Neurology, USA). These neurophysiological examination tests were performed in our hospital by staff in the electrophysiology room for medical use. The information of motor conduction fiber velocity of the ulnar nerve, radial nerve, median nerve, tibial nerve, and common peroneal nerve, as well as the sensory conduction fiber velocity of the ulnar nerve, radial nerve, median nerve, and sural nerve, was collected from the medical histories of patients.

### 2.5. Sample Size Calculation

The minimum sample size was calculated by PASS V11.0.7 (NCSS, USA). Before we finished the work of volunteer recruitment, we had estimated the minimum sample size by mean and standard deviation of SUA (data from recruited patients at that time). When we finished the volunteer recruitment work, we confirmed that the sample size is sufficient. Minimum sample sizes of patients without DPN and those with DPN are 48 and 56, respectively, according to the data from all recruited patients.

### 2.6. Statistical Methods

Data was analyzed by SPSS 22.0 (IBM, USA). *T*-tests were carried out to compare the difference of normally distributed variables in patients with and without DPN. Mann–Whitney *U* Tests were performed to compare the difference of asymmetrically distributed variables in the control group and the DPN group. Chi-squared tests were applied to compare the difference of the binary variable in the two groups. Pearson's correlation, partial correlation analysis, binary logistic regression analysis, and multiple linear regression analysis were performed to explore the relationships between DPN and SUA. *p* < 0.05 was defined as statistical significance. The methods (including statistical methods and some other descriptions) are similar with our previous published manuscript [31].

## 3. Results

### 3.1. Clinical Parameter Result of T2DM Patients with and without DPN

To explore the potential risk factors of DPN in patients with T2DM, baseline data of diabetic patients with DPN and those without DPN were compared. As shown in Table 1, there was no significant difference in age, gender, BMI, duration of diabetes, duration of hypertension, TG, TC, LDL-C, and HDL-C in T2DM patients with DPN and those without DPN (all *p* > 0.05). While increased HbA1c was found in 57 patients with DPN, decreased SUA was detected in patients with DPN, compared to 49 diabetic patients without DPN (All *p* < 0.05) (Table 1).

### 3.2. Neurophysiological Examination Result of T2DM Patients with and without DPN

In this present work, we compared the difference in motor conduction fiber velocity of the ulnar nerve, radial nerve, median nerve, tibial nerve, and common peroneal nerve, as well as the sensory conduction fiber velocity of the ulnar nerve, radial nerve, median nerve, and sural nerve patients. Compared with 49 patients in the control group, patients in the DNP group showed not only decreased motor conduction fiber velocity of the ulnar nerve, radial nerve, median nerve, tibial nerve, and common peroneal nerve but also impaired sensory conduction fiber velocity of the ulnar nerve, radial nerve, median nerve, and sural nerve (all *p* < 0.05) (Table 2).

### 3.3. Pearson's Correlation between SUA and Nerve Conduction Velocity

To confirm the relationship between SUA and DPN in patients with T2DM, Pearson's correlation was conducted. Here, it is demonstrated that SUA is not only associated with tibial nerve motor conduction velocity (*R* = 0.247, *P* = 0.011) but also related to median nerve (*R* = 0.211, *P* = 0.030) and sural nerve (*R* = 0.223, *P* = 0.022) sensory conduction velocity in T2DM patients (Table 3).

### 3.4. Partial Correlation between SUA and Nerve Conduction Velocity Adjusted for HbA1c

As HbA1c levels are higher in patients with DPN than those without DPN, partial correlation analysis was carried out and adjusted for HbA1c to further explore the relationship between SUA and DPN in diabetic patients without uric acid lowering drugs. It is different from the results of Pearson's correlation. We found an association between SUA and tibial nerve motor conduction velocity (*R* = 0.197, *p* = 0.044), rather than median nerve and sural nerve sensory conduction velocity (all *p* > 0.05) (Table 4).

### 3.5. Comparison of SUA Levels in Different Patients with T2DM

**Figure 1:**
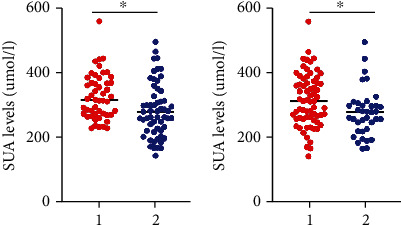
SUA levels in different patients with T2DM. ^∗^*p* < 0.05. “1” in (a) showed T2DM patients without DPN; “2” in (a) showed T2DM patients with DPN; “1” in (b) showed T2DM patients without decreased tibial nerve motor conduction velocity; “2” in (b) showed T2DM patients with decreased tibial nerve motor conduction velocity. Abbreviations: T2DM: type 2 diabetes mellitus; SUA: serum uric acid.

As shown in the baseline data, decreased SUA was found in diabetic patients with DPN, compared to those without DPN. Here, we showed this result in a scatter diagram (Figure 1(a)). The above result indicated that SUA is associated with tibial nerve motor conduction velocity. We also compared the difference in SUA levels between T2DM patients with or without tibial nerve motor fiber damage. Interestingly, we also found decreased SUA levels in patients with slow tibial nerve motor conduction velocity (Figure 1(b)).

### 3.6. Low SUA Level Is One of the Risk Factors of DPN in T2DM Patients

To further investigate the role of SUA in diabetic patients with DPN, binary logistic regression analysis was conducted and adjusted for HbA1c. It is showed that lower SUA is one of the risk factors of DPN in T2DM patients independent from HbA1c (OR = 0.994, *p* = 0.043) (Table 5).

### 3.7. Low SUA Levels May Influence Tibial Nerve Motor Conduction Velocity

To further investigate the effect of SUA levels on the details of DPN in patients with T2DM patients, multiple linear regression analysis was performed. After adjusting for HbA1c, low SUA may influence the tibial nerve motor conduction velocity of diabetic patients with DPN (*β* = 0.012, *p* = 0.044) (Table 6).

## 4. Discussion

DPN is one of the most important complications of diabetes associated with hyperglycemia [32, 33]. Indeed, increased HbA1c levels were detected in patients with DPN, compared with those without DPN. It is agreed with a previous study [34]. Except for chronic hyperglycemia, oxidative stress may also be involved in the development of DPN. Indeed, oxidative stress plays an important role in painful diabetic peripheral neuropathy [23]. Additionally, the occurrence of oxidative stress may also result from hyperglycemia [35]. Moreover, it demonstrated the relationship between oxidative stress and DPN in humans [24], animals [26, 36], and in vitro [37] studies. As described in the introduction, SUA may be involved in DPN, which is associated with oxidative stress [38–40]. We also compared with levels of SUA, which is a kind of natural antioxidant [28], in T2DM patients with or without DPN. Interestingly, decreased SUA was observed in diabetic patients with DPN. Similarly, a previous study showed lower SUA in T2DM patients with mild cognitive impairment than those without cognition decline [41], which is a kind of dysfunction in the central nervous system [42, 43]. To confirm the occurrence of DPN, the conduction velocity of nerve was compared. Undoubtedly, motor conduction fiber velocity of the ulnar nerve, radial nerve, median nerve, tibial nerve, and common peroneal nerve as well as sensory conduction fiber velocity of ulnar nerve, radial nerve, median nerve, and sural nerve was faster in patients without DPN than those with DPN.

Due to the decreased SUA levels in patients with DPN, Pearson's correlation was conducted to detect the association between SUA and the above conduction velocity of nerves. We found that levels of SUA were positively associated with the conduction speed of tibial nerve motor fibers, median nerve sensory fibers, and sural nerve sensory fibers. Additionally, we described the increased HbA1c levels in DPN patients. Here, we performed a partial correlation adjusted for HbA1c, to further investigate the association between SUA and DPN. SUA was associated with tibial nerve motor conduction velocity adjusted for HbA1c in this present study.

After adjusting by HbA1c, SUA was associated with tibial nerve motor conduction velocity in patients with T2DM. So, not only the levels of SUA in diabetic patients with and without DPN but also those in patients with normal and decreased tibial nerve motor conduction velocity were observed. Both decreased SUA levels in DPN patients and patients with decreased tibial nerve motor conduction velocity were found.

To confirm the risk factor of DPN in patients with T2DM, binary logistic regression analysis was conducted and adjusted for HbA1c. It is showed that lower SUA is one of the risk factors of DPN in T2DM patients independent from HbA1c. To further investigate the relationship between SUA and nerve injury details, multiple linear regression analysis was performed by adjusting for HbA1c. Decreasing SUA may influence the tibial nerve motor conduction velocity of diabetic patients with DPN. It is demonstrated that low SUA levels are associated with DPN. However, increased SUA levels are also associated with the occurrence of DPN in T2DM patients [17]. These contradictions may result from different populations included. In this present study, we included hospitalized patients without SUA lowering treatments. Patients with high levels of SUA were excluded due to the use of SUA lowering drugs. The neuroprotective effect of antioxidant SUA may be offset in patients with abnormally increased SUA levels and other metabolic disorders associated with hyperuricemia. As a kind of metabolic disorder, extremely high levels of SUA may lead to an increased risk of DPN along with other factors of metabolic disorder [19].

Generally, previous research has mainly investigated the risk factors associated with DPN in individuals with diabetes. The study investigated the characteristics of T2DM patients with and without peripheral neuropathy, focusing particularly on nerve conduction velocities and SUA levels. The present study, however, not only explored the risk factors for DPN but also examined the association between SUA levels and specific neuronal damage in patients with T2DM. Nonetheless, there are several limitations that require addressing. Firstly, the study design was cross-sectional, thus allowing for the establishment of only an association rather than a causal relationship between SUA and DPN. Secondly, uric acid-lowering drugs were used as an essential factor in uric acid disorder treatment. Here, patients with uric acid-lowering drugs were excluded in this research. So, the analysis of the kind and dosage of uric acid-lowering drug treatment was not sufficient in this study. Lastly, the study only considered the effect of decreased SUA levels on DPN, neglecting the potential impact of elevated SUA levels.

## 5. Conclusion

To the best of our knowledge, this is the first study focusing on the relationship between the DPN, especially for certain nerve conduction velocities, and SUA in T2DM patients without uric acid lowering treatments. We demonstrated that low SUA is a risk factor for DPN in patients with T2DM. Additionally, decreased SUA may influence the function of the tibial nerve motor fiber independent from the control of HbA1c. This may result from the antioxidant effect of SUA. Increasing SUA to a certain level may be a novel method to reduce the burden of DPN in T2DM patients. Further well-designed prospective cohort studies and basic researches are needed to clarify the causal association between SUA and DPN as well as the mechanisms associated with oxidative stress of SUA in DPN of T2DM patients.

## Figures and Tables

**Table 1:** Comparation of clinical parameters between control and DPN group.

	Control group (*n* = 49)	DPN group (*n* = 57)	*p*
Age (years)	55.43 ± 10.59	56.02 ± 13.66	0.807^a^
Female (*n*, %)	17, 34.7	23, 40.4	0.177^c^
BMI (m^2^/kg)	24.61 (22.57-26.38)	24.30 (21.62-26.53)	0.606^b^
Duration of DM (years)	7 (3-10)	7 (1-15)	0.610^b^
Duration of HBP (years)	0 (0-7)	0 (0-4.5)	0.840^b^
HbA1c (%)	8.80 (6.85-9.85)	10.6 (9.20-12.15)^∗^	0.001^b^
TG (mmol/l)	1.75 (1.26-2.49)	1.42 (1.07-2.20)	0.194^b^
TC (mmol/l)	4.54 (3.84-5.12)	4.47 (3.95-5.10)	0.917^b^
HDL-C (mmol/l)	0.85 (0.67-1.07)	0.84 (0.69-1.11)	0.646^b^
LDL-C (mmol/l)	2.38 (1.69-2.79)	2.32 (1.92-3.19)	0.219^b^
Scr (umol/l)	63.00 (49.50-72.50)	58.00 (49.50-72.00)	0.375^b^
BUN (mmol/l)	5.84 (5.15-6.84)	5.74 (4.97-7.53)	0.807^b^
SUA (mmol/l)	326.27 ± 69.79	284.24 ± 83.17^∗^	0.006^a^

The data are presented as *n* (%) or the median (interquartile range) unless otherwise specified. ^a^Student's *t*-test was employed for normally distributed variables. ^b^The Mann–Whitney *U* test was employed for asymmetrically distributed variables. ^c^The Chi-square test was employed for categorical variables. ^∗^*p* < 0.05, DPN group vs. control group. Abbreviations: DPN: diabetic peripheral neuropathy; BMI: body mass index; DM: diabetes mellitus; HBP: high blood pressure; TG: triglycerides; TC: total cholesterol; LDL-C: low-density lipoprotein cholesterol; HDL-C: high-density lipoprotein cholesterol; Scr: serum creatinine; BUN: blood urea nitrogen; SUA: serum uric acid.

**Table 2:** Comparation of neurophysiological test results between control and DPN group.

	Control group (*n* = 49)	DPN group (*n* = 57)	*p*
*Motor conduction*			
Ulnar nerve (m/s)	61.95 (60.05-63.15)	54.60 (51.85-60.68)^∗^	<0.001^b^
Radial nerve (m/s)	64.85 (62.60-66.53)	63.55 (61.25-65.68)^∗^	0.014^b^
Median nerve (m/s)	60.20 (56.93-62.20)	54.25 (50.50-56.50)^∗^	<0.001^b^
Tibial nerve (m/s)	47.37 ± 3.10	40.95 ± 4.17^∗^	<0.001^a^
Common peroneal nerve (m/s)	46.75 (45.13-48.68)	43.45 (40.03-44.98)^∗^	<0.001^b^
*Sensory conduction*			
Ulnar nerve (m/s)	56.70 (52.33-60.50)	49.00 (43.23-53.43)^∗^	<0.001^b^
Radial nerve (m/s)	57.79 ± 8.41	49.82 ± 5.96^∗^	<0.001^a^
Median nerve (m/s)	58.15 (54.00-62.50)	45.55 (40.48-51.55)^∗^	<0.001^b^
Sural nerve (m/s)	52.35 ± 5.03	44.63 ± 5.48^∗^	<0.001^a^

^a^Student's *t*-test was employed for normally distributed variables. ^b^The Mann–Whitney *U* test was employed for asymmetrically distributed variables. ^∗^*p* < 0.05, DPN group vs. control group. Abbreviations: DPN: diabetic peripheral neuropathy.

**Table 3:** Pearson's correlation between SUA and nerve conduction velocity.

	Pearson correlation (*R*)	*p*
*Motor conduction*		
Ulnar nerve	0.144	0.142
Radial nerve	-0.026	0.094
Median nerve	0.178	0.067
Tibial nerve	0.247	0.011^∗^
Common peroneal nerve	0.097	0.321
*Sensory conduction*		
Ulnar nerve	0.131	0.182
Radial nerve	0.151	0.123
Median nerve	0.211	0.030^∗^
Sural nerve	0.223	0.022^∗^

^∗^ *p* < 0.05. Abbreviations: SUA: serum uric acid.

**Table 4:** Partial correlation between SUA and nerve conduction velocity adjusted for HbA1c.

	Partial correlation (*R*)	*p*
*Motor conduction*		
Ulnar nerve	0.090	0.360
Radial nerve	-0.040	0.684
Median nerve	0.144	0.144
Tibial nerve	0.197	0.044^∗^
Common peroneal nerve	0.023	0.820
*Sensory conduction*		
Ulnar nerve	0.090	0.361
Radial nerve	0.098	0.318
Median nerve	0.160	0.103
Sural nerve	0.159	0.104

^∗^ *p* < 0.05. Abbreviations: SUA: serum uric acid.

**Table 5:** Binary logistic regression analysis for risk factors of DPN in T2DM patients.

	Binary logistic regression (OR)	95% CL of OR		*p*
HbA1c	0.348	1.112	1.633	0.002^∗^
SUA	0.994	0.989	1.000	0.043^∗^

^∗^ *p* < 0.05. Abbreviations: DPN: diabetic peripheral neuropathy; T2DM: type 2 diabetes mellitus; SUA: serum uric acid.

**Table 6:** Multiple linear regression analysis for factors that influence the tibial nerve motor conduction velocity of T2DM patients.

	Multiple linear regression (*β*)	95% CL of *β*		*p*
HbA1c	-0.496	-0.839	-0.100	0.013^∗^
SUA	0.012	≤0.001	0.023	0.044^∗^

^∗^ *p* < 0.05. Abbreviations: T2DM: type 2 diabetes mellitus; SUA: serum uric acid.

## Data Availability

All data in this manuscript have been submitted to our institute for records. Additionally, all IDs of recruited patients were also submitted for further use. The datasets analyzed are available from authors on reasonable request.
